# Surgery Notices

**Published:** 1863-06

**Authors:** 


### SURGERY NOTICES.

Many applications are made to us, from time to time, to know where may be obtained safe, skillful and competent surgical aid. In all operations requiring undisputed professional ability, a keen eye, a steady hand and a comprehensive grasp of intellect, we believe there are few superior to Dr. Henry A. Daniels, of New-York City, author of a treatise on the Fifth Pair of Nerves and several other anatomical and surgical monograms; a gentleman of whose professional ability, skill and coöperation, several of the most eminent practitioners in this country have greatly availed themselves, at various times, and of whose success in the removal of tumors, in correcting deformities of face, cheek, lips, eyes, and person; strabismus, (cross-eyes;) in rectifying misplacements, curing leucorrheas, strictures, piles, fistulas, and all important surgical cases, many patients will gladly testify. Any of our readers who will place themselves in the hands of this talented consulting and operating surgeon, may rest assured that whatever a skillful surgery could do for them, will be happily done by Dr. Daniels. A grateful soldier, cured of an injury received in camp, writes from Washington City: "It will always give me great pleasure to testify to Dr. D.'s ability as an operative surgeon." The New-York *Independent*, very chary of its praises, says in its issue of January 22d, 1863: "Doctor H. A. Daniels, late Professor of Surgery in the Pennsylvania Medical Hospital of Philadelphia, but now of 221 Sixth Avenue, New-York, has become justly renowned for skillful and successful treatment of some of the sorest ills 'that flesh is heir to.' Let all who are afflicted with the grievous maladies which it is his peculiar province to cure, and who have suffered at the hands of ignorant pretenders, or by use of vulgar nostrums, lose no time in availing themselves of the advantages which advanced science now holds out to them."

The following notice is copied from the New-York *Independent*:

I insert this testimonial for the purpose of publicly returning my most sincere thanks to Dr. H. A. Daniels, of 221 Sixth Avenue, *for his skill in reproducing for me the lower portion of my nose*, which I lost by an injury some years ago.

Anne Murphy,

547 Eighth Avenue, N. Y.

*From the Philadelphia Mercury*

Surgical Operation.—On Thursday last an enormous steatomatous tumor was cut from the back of Mrs. Davis, of Thirteenth street, by H. A. Daniels, M.D.—The wen or sebaceous cyst weighed over four pounds. Dr. Daniels was assisted by Dr. S. Pancoast in the operation, in the presence of A. P. Thomas, M.D., J. S. Longshore, M.D., and Hannah Longshore, M.D. The lady is doing extremely well, and rejoices in the removal of the abnormal difficulty.

*From the New-York Herald, Dec.* 18, 1862.

I hereby return my thanks to Dr. H. A. Daniels, 221 Sixth Avenue, near Fourteenth street, for skillfully removing, without pain, a large foreign substance from my ear, which had troubled me for a considerable time. I can fully testify to the Doctor's ability as a surgeon.

G. Boweryem,
132 Bleecker Street.

A Missionary at Corisco, West-Africa, writes: "I received but a few days ago a bound volume of Hall's Journal of Health. I thank you for it. I have looked through it with pleasure. I have occasionally seen numbers of your publications when in my native land. I certainly wish you great success in inculcating the principles which your JOURNAL advocates. With prayers that you may enjoy many years of usefulness, I remain sincerely yours." A correspondent says of our book on "Sleep": "I have read and profited by it. Many thanks for its teachings, and God bless you for writing it." A lady in ordering some copies of "Soldier Health" for the army, adds: "I hope it is not abridged too much, for it is too good to leave any thing out. I wish to send a copy to many in the army."

THE AMERICAN TRACT SOCIETY, 28 Cornhill, Boston, and 13 Bible-House, New-York City, has issued in beautiful style, "Calls to the Saviour," (64 pp.,) or reasons why every sinner should "Come to Jesus." It is full of comfort and encouragement to those who are seeking a "better way." Another little volume of 128 pp., by Rev. James D. Burns, of London, is full of sweetness to every hoping Christian heart. "The Happy Home," or the Story of Annie Lyon, with its illustrations, will gladden the eyes and swell the heart of every good child who reads it. We congratulate the Society in its issue of two useful, truthful books. In "The Honey-Makers" and "The Senses," both children and grown persons will be deeply interested; and more, they will be led to admire the wisdom and goodness of God while they peruse these two charming books. Is the Society beginning to find out that a deeper religious sentiment can be waked up in the minds of the young by showing to them the wonders of Omnipotence in his works, than by the trashy narration of the vagaries of a maudlin imagination; by stories one part truth, nine hundred and ninety-nine parts "make ups"? A narration of actual facts, in a natural, literal way is always instructive, is a "True Story," but such a book can not be found oftener perhaps than once in a hundred volumes of the publications designed for the young, and for Sunday-schools. Ought this so to be? "Kenny Carl's Uniform" is as instructive as it is interesting, and will delight youthful readers.

TO SUBSCRIBERS.—A lady, in writing for a missing number, not only expects to have it supplied at our own cost, but growls while receiving it, saying: "I thought my responsibilities ended with paying for the JOURNAL." Let subscribers remember that all a publisher's obligations end with depositing the publication properly in the post-office. Publishers do not agree to deliver their issues at the door or into the hand of subscribers out of town, but merely to deposit them in the post-office, then the government takes the responsibility, and the subscriber the risk. Publishers, generally, out of mere good-will, supply missing numbers, but it is their own loss. The intelligently conscientious will send the money for missing numbers, for when they subscribe it is with the understanding that the publication is to be sent by mail, and the subscriber tacitly agrees to run the risk, but afterwards to ask the publisher to make good the default is simply unjust. The usual manner of mailing publications, makes it *difficult to omit any name.* Persons writing to us must not ask for receipts to be returned, the reception of this JOURNAL is a receipt, for it is never sent without pay in advance. ☞ All subscriptions must be for a year, and the year must commence with the preceding January.

